# Superconducting Properties and Electron Scattering Mechanisms in a Nb Film with a Single Weak-Link Excavated by Focused Ion Beam

**DOI:** 10.3390/ma14237274

**Published:** 2021-11-28

**Authors:** Marlon Ivan Valerio-Cuadros, Davi Araujo Dalbuquerque Chaves, Fabiano Colauto, Ana Augusta Mendonça de Oliveira, Antônio Marcos Helgueira de Andrade, Tom Henning Johansen, Wilson Aires Ortiz, Maycon Motta

**Affiliations:** 1 Departamento de Física, Universidade Federal de São Carlos, São Carlos 13565-905, SP, Brazil; marlon190@gmail.com (M.I.V.-C.); davi@df.ufscar.br (D.A.D.C.); fcolauto@df.ufscar.br (F.C.); wortiz@df.ufscar.br (W.A.O.); 2 Instituto Federal de Educação, Ciência e Tecnologia de São Paulo, Campus São Carlos, São Carlos 13565-905, SP, Brazil; oliveira.a.a.m@gmail.com; 3 Instituto de Física, Universidade Federal do Rio Grande do Sul, Porto Alegre 91501-970, RS, Brazil; antonio.andrade@ufrgs.br; 4 Department of Physics, University of Oslo, POB 1048, Blindern, NO-0316 Oslo, Norway; t.h.johansen@fys.uio.no

**Keywords:** granular superconductivity, weak-link, thin film, ion implantation

## Abstract

Granularity is one of the main features restricting the maximum current which a superconductor can carry without losses, persisting as an important research topic when applications are concerned. To directly observe its effects on a typical thin superconducting specimen, we have modeled the simplest possible granular system by fabricating a single artificial weak-link in the center of a high-quality Nb film using the focused ion beam technique. Then, its microstructural, magnetic, and electric properties in both normal and superconducting states were studied. AC susceptibility, DC magnetization, and magneto-transport measurements reveal well-known granularity signatures and how they negatively affect superconductivity. Moreover, we also investigate the normal state electron scattering mechanisms in the Boltzmann theory framework. The results clearly demonstrate the effect of the milling technique, giving rise to an additional quadratic-in-temperature contribution to the usual cubic-in-temperature *sd* band scattering for the Nb film. Finally, by analyzing samples with varying density of incorporated defects, the emergence of the additional contribution is correlated to a decrease in their critical temperature, in agreement with recent theoretical results.

## 1. Introduction

Structured thin films have shown promise for applications in different areas, including electronics and medicine. In particular, micro- and nanoscopic patterned superconducting films have received attention as their electric and magnetic properties may be optimized for technological purposes [1,2,3,4,5,6,7,8,9,10,11,12,13]. In this context, focused ion beam (FIB) milling is an extraordinary tool that allows to obtain structures not possible to design by other nanofabrication methods [14,15,16]. FIB can reach spatial accuracy of about 20 nm and has been used to control the superconducting properties of several materials, including niobium films [4,6,8,10,13,17,18,19,20]. A serious drawback, however, is an inevitable contamination due to ion implantation that may cause severe damage to the milled material, modifying its physical characteristics [15,21] and being detrimental to superconducting specimens [22,23]. It has been reported for Nb films [24] that FIB leads to gallium implantation resulting in a systematic increase in their residual resistivity accompanied by a decrease in the superconducting critical temperature ($T_{c}$). Such a behavior is also common to other ionic specimens, as well as implantation techniques [25,26,27].

It is well-known that the temperature-dependent resistivity ρ(T) of most metals can be modeled as a power law, $\rho \left(T\right)\propto T^{n}$, where the exponent *n* is governed by interactions between electrons and lattice vibrations [28]. In general terms, for temperatures above a material-dependent threshold that can usually be approximated by the Debye temperature $\Theta_{D}$, ρ(T) increases linearly as the scattering mechanism is related only to fluctuations of the metallic ions about their equilibrium lattice sites [28]. For $T<\Theta_{D}$, it is important to account for lattice motion and electron-phonon interactions to properly describe the electronic scattering, whose probability to occur is greatly reduced. This, suitably, diminishes the electrical resistance leading to an n=5 behavior for regular metals according to the Bloch-Grüneisen description [29]. For transition metals, such as Nb, the picture is different due to an overlap between the upper filled *s*-band and the partially unfilled *d*-band with drastically different Fermi velocities that may act as traps, removing scattered electrons from the conduction band [30]. Between $\Theta_{D}$ and a certain temperature proportional to the minimum phonon momentum required to scatter electrons from the *s*- to the *d*-band, Wilson [31] showed that such *sd* interband scattering leads to a cubic-in-*T* (n=3) behavior of ρ(T), which was also verified experimentally [32].

On the other hand, the inclusion of impurities in the crystalline structure, which occurs during FIB milling due to ionic implantation, is expected to influence the normal state transport properties of superconducting materials [33]. In this case, a new n=2 additive contribution to ρ(T) is present. Although somewhat different approaches have been used to explain such term–see, for instance, Refs. [34,35]–the main idea is that there is an enhancement in electron-electron scattering due to interactions with lattice defects [36]. A recent work sheds new light on the nature of the relationship between superconductivity and the emergence of this quadratic-in-*T* normal state resistivity behavior for thin films [37]. By combining the analysis of several experimental data and different theoretical techniques, the authors unveiled the role of lattice distortion and softening in establishing a so-called pseudo-Umklapp electron-electron scattering channel. Their approach led to analytical expressions for $T_{c}$ and the quadratic-in-*T* coefficient dependency on the residual resistivity, depending on the electron-phonon coupling regime and a universal kinematic scaling relationship.

Concerning practical applications of type-II superconductors, an ingredient must be considered: the presence of weak-links (WLs) [38]. Many superconducting materials are inherently comprised by arrangements of two distinctive regions: grains, which present bulky superconductivity, and WLs, at which the superconducting properties are partially or completely suppressed. For instance, these weak-link regions can be junctions of conducting, insulating, or weaker superconducting materials between neighboring grains [38,39], misaligned grain boundaries in polycrystalline samples [40], areas penetrated by magnetic fields [41], and nonstoichiometric material among the grains – a prominent feature in ceramic specimens [42]. Although WLs are essential to some superconducting devices, most notably SQUIDs that take advantage of the Josephson Effect to measure magnetic fields with unmatched accuracy [43], their presence deteriorates the overall connectivity of the grains as a result of different inter- and intragrain critical current densities [42]. Effectively, they depreciate the maximum supercurrent which the superconductor is able to transport, representing a rather limiting consequence for certain applications. An important example is the natural occurrence of granularity in high-temperature cuprate superconductors, for which the improvement of transport properties has been a key topic of study along the last four decades [40,44,45,46,47]. For such materials, the decrease of the critical current in a single grain boundary has been demonstrated experimentally [48,49] by means of transport measurements and magneto-optical Imaging (MOI) of bicrystalline YBa${}_{2}$Cu${}_{3}$O${}_{7-\delta }$ thin films. In this case, single-crystalline superconducting grains are connected by an intergrain region or a single grain boundary. On the other hand, the presence of macroscopic defects created artificially in low-temperature superconducting thin films, such as in patterned or ion-implanted Nb devices [18,50], may create areas of suppressed superconductivity which separates large superconducting regions. These regions behave approximately as extended macroscopic superconducting grains despite being composed of several interconnected single crystalline grains, i.e., a polycrystalline material. Therefore, we borrow the terminology *grain* for this extended superconducting grain hereafter. For the latter group of samples, the FIB technique stands out once more, since it does not only allow patterning samples with great precision, but the resulting ionic implantation is expected to degrade the superconducting properties.

In this work, we present a toy model aiming to investigate the influence of a single WL in both superconducting and normal state properties of a high-quality niobium thin film. Our sample is prepared by excavating a nanoscale-depth groove along the width of the rectangular film using FIB milling, resulting in a system comprising two superconducting grains connected by a WL, i.e., the groove. Magnetic responses are investigated via AC susceptibility and DC magnetization measurements for different applied magnetic field and temperature configurations, evidencing the granular signatures of the specimen. Additionally, we studied the normal state electronic transport in the sample using resistivity measurements for one grain, for the groove, and for the series association of the two grains and the groove. These results were analyzed in the framework of the Boltzmann theory, considering the Bloch-Grüneisen and Wilson regimes, and reveal the expected n=3 behavior for the niobium grains. In the impurity-rich groove region, resistivity data demonstrate the presence of an additional n=2 term in ρ(T), which is directly connected to the milling process. Finally, it is possible to obtain the electronic mean free path from the transport data for both contributions and calculate two typical length scales related to the superconducting behavior, the coherence length and the penetration depth, once more displaying a deterioration of the superconductivity due to the presence of the weak-link.

## 2. Materials and Methods

**Figure 1:**
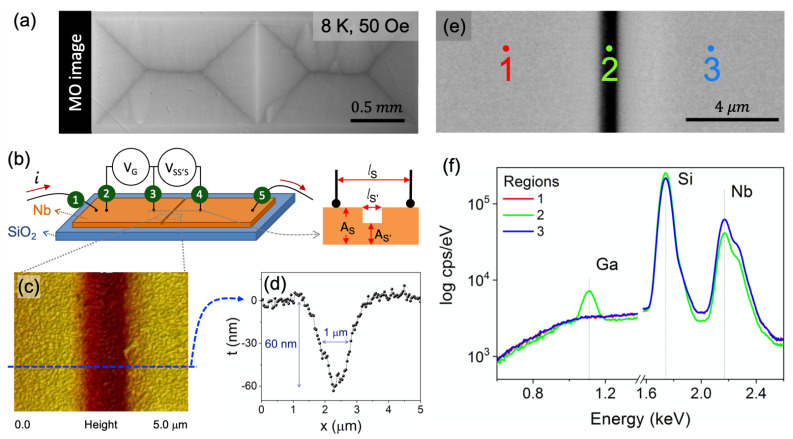
(Color online) (**a**) Magneto-optical (MO) image of the grooved Nb film taken at 8 K and 50 Oe after a ZFC procedure. (**b**) Schematic representation of the sample and details of the contact leads used in transport measurements. (**c**) AFM image and (**d**) profile for the SS′S region. (**e**) SEM image and the identification of three different points at which the EDS spectra were taken. (**f**) EDS spectra showing the $K_{\alpha }$ line for gallium, which appears only in the region inside the groove (2).

A 180 nm thick Nb film was deposited onto a SiO${}_{2}$ substrate by magnetron sputtering under base pressure lower than 2 × 10${}^{-8}$ Torr. Standard optical lithography was used to sharply define the borders of a rectangular film with 3 × 1 mm${}^{2}$. The tailored WL was artificially created by the excavation of a micrometric-width groove prepared in a FIB apparatus by adjusting a dose of 1.2 nC/μm${}^{2}$. The groove crossed the entire extension of the central region of the film perpendicularly to the longer edges, creating thus two identical regions with the same superconducting properties (S) coupled by a less robust superconductor (S′), as exemplified in Figure 1a,b. For comparison, a non-patterned control sample, i.e., a pristine film, was also prepared. Additionally, to investigate the relationship between $T_{c}$ and the disorder caused by the milling procedure, five different samples were prepared analogously by changing the beam dose to 0.1, 0.2, 0.3, 0.6, and 1.8 nC/μm${}^{2}$.

AC susceptibility measurements $\chi_{AC}\left(T\right)=\chi^{\prime }\left(T\right)+i\chi^{\prime \prime }\left(T\right)$ were carried out with a commercial Quantum Design MPMS-5S (Quantum Design, San Diego, USA). The real in-phase component $\chi^{\prime }$ is associated with the inductive response, i.e., the magnetic moment, whereas the imaginary out-of-phase part χ" refers to the resistive response of the superconductor, i.e., its resistive losses caused by the oscillating excitation field [51,52,53]. $\chi_{AC}\left(T\right)$ was obtained for temperatures ranging from 2K up to 10K, using a fixed frequency *f* of 100Hz, for values of the excitation field within the ±3.8Oe interval. Temperature-dependent DC magnetization curves were obtained using the same setup for T= 6.0, 6.5, and 7.0 K under applied DC magnetic fields in the range ±500Oe. Both AC and DC fields were always applied perpendicular to the plane of the film.

The topography of the film was investigated by atomic force microscopy (AFM) in a Digital Instruments Nanoscope V (Digital Instruments, Santa Barbara, CA, USA). The images were obtained in the peak-force tapping mode. The cantilevers used were Veeco Antimony(n)-doped Si model TAP150A and their tip was shaped like a polygon-based pyramid, with radius of 8 nm, height of 17.5 μm and tip set back of 15 μm. Besides that, the sample composition was inspected using an Energy Dispersive X-ray Spectrometry (EDS) module coupled to a Philips XL-30 FEG Scanning Electron Microscope (FEI Company, Eindhoven, The Netherlands) (SEM), which was also employed to obtain complementary information on the film surface. The acceleration voltage used was 15 kV and the diameter of the probe was 500 nm. The estimated statistical error in the elemental composition is 3.5%.

To provide electric contacts, five aluminum wires with diameter of 20 μm were inserted on the film by a TPT-HB05 Wirebonder (TPT, Munich, Germany) in the wedge mode. Figure 1b shows a scheme of the electric contacts placed in the plain region and in the excavated groove. An electric current was applied between the electrodes identified as 1 and 5, and the voltage was measured in two different channels, between the electrodes 2 and 3 for the plain region (V${}_{G}$) and, separately, between the electrodes 3 and 4 for the excavated region ($V_{\mathrm{SS}\prime S}$), resulting in the standard four-probe configuration for each contribution. Thus, it was possible to compare the transport properties of the unspoiled part of the film with the one etched by the FIB.

Transport measurements were carried out in a Quantum Design PPMS-6000 (Quantum Design, San Diego, CA, USA) to determine the superconducting and normal state parameters associated with the grains and the weak-link. In order to obtain the WL resistivity, the different sections of the sample were treated as resistors in series. The resistance between the electrodes 3 and 4 is due to a plain region (S), summed to the grooved region (S′), and the second plain region (S). Thus, the resistivity due only to the groove $\rho_{S^{\prime }}$ is given by:

(1) $$\rho_{S^{\prime }}=\left(\frac{A_{S^{\prime }}}{l_{S^{\prime }}}\right)\cdot \left(R-\frac{\rho_{G}\left(l_{S}-l_{S^{\prime }}\right)}{A_{S}}\right)$$

where $\rho_{G}$ is the grain resistivity, which is equivalent to the S contribution. $A_{S}$, $A_{S^{\prime }}$, $l_{S}$, and $l_{S^{\prime }}$ are the cross-section areas and the lengths of the S and S′ contributions, respectively. Although the groove presents a non-uniform width (see Figure 1d), the resistivity results were obtained by considering its profile as a right angle step, as sketched in Figure 1b.

## 3. Results

This section is divided into four parts: (i) Sample characterization; (ii) Superconducting properties; (iii) Normal state properties; and an analysis of (iv) The evolution of the critical temperature with disorder, which are described ahead.

### 3.1. Sample Characterization

Concerning the terminology “grain” to define the polycrystalline unpatterned Nb film regions, one should be careful to inspect if, indeed, both areas behave uniformly as unique superconducting entities. A powerful technique to do so is MOI, which allows one to map the flux distribution [54,55]. Figure 1a shows a magneto-optical (MO) image for the grooved Nb specimen captured using MOI at 8 K for an applied field of 50 Oe after a zero field cooling (ZFC) procedure. The MO image demonstrates that the two large superconducting regions separated by the groove are independently filled by magnetic flux. Besides that, the groove presents a higher gray intensity, i.e., a higher intensity of magnetic flux penetrates the groove, as a consequence of weaker shielding currents. Thus, the flux penetration patterns are similar to those obtained by Palau et al. [56] and Polyanskii et al. [49] for YBCO films grown on bicrystal substrates, i.e., two grains separated by a grain boundary. Therefore, it is reasonable to treat our grooved sample as a system comprising two superconducting grains, separated by a single weak-link.

An important step to ensure that the studied specimen reproduces properly the proposed system is to inspect the quality of the notch along the sample width after patterning. For this, a careful analysis has been done throughout the groove and its surroundings using AFM, SEM, and EDS. A representative AFM image of this region is presented in Figure 1c, which shows a top view of the center of the specimen. A cross-section scan along the dashed line is shown in panel (c). The groove shows a V-shaped profile with full-width at half-height and depth of approximately 1.0μm and 60nm, respectively. A SEM image, presented in Figure 1e, also confirms this width.

In order to determine its final composition and the existence of Ga implanted into the groove, three different sample regions were mapped using EDS spectra. Figure 1e shows schematically the position where the spectra were taken: one exactly at the notch, labeled as 2, and other two in the plain parts of the Nb film, on each side of the channel, labeled as 1 and 3. The spectra are presented in Figure 1f for those regions, exhibiting peaks related to Si and Nb from the substrate and the superconducting film, respectively. As expected, regions 1 and 3 show quite similar results. Nonetheless, an additional peak appears at 1.11keV at the channel position, being identified as due to gallium implanted during excavation. The composition in position 2, considering only Nb and Ga elements, is Nb-5.3at%Ga. Another evidence of the thickness variation in the sample is given by the peak height of the different elements in the EDS spectra. The Nb peak is smaller for the groove, whereas the peak related to Si is larger in comparison to regions 1 and 3. It occurs due to the thinner thickness of Nb in the groove, resulting in a deeper interaction range with Si when compared to the plain Nb regions. The analysis confirms that, as designed, the sample is composed of two Nb superconducting grains connected by a region of expected depressed superconductivity, i.e., the WL.

### 3.2. Superconducting Properties

The superconducting properties were investigated by using different techniques. AC susceptibility, DC magnetization, and magneto-transport measurements were employed to characterize the consequences of grooving the Nb film using FIB.

**Figure 2:**
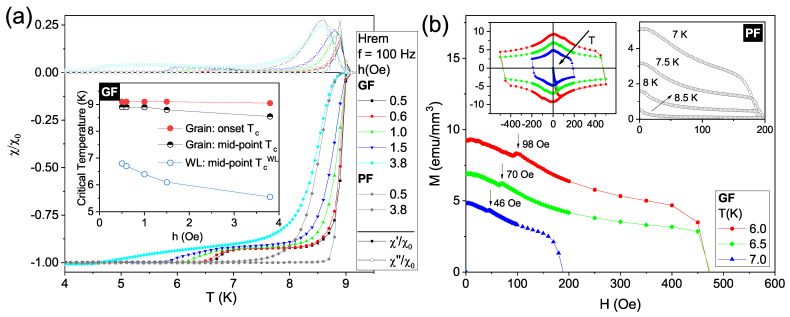
(Color online) (**a**) Temperature-dependent AC susceptibility for two different samples: a plain film (PF) and a structured film with a central groove (GF). The measurements were taken for 100Hz and remnant DC field ($H_{\mathrm{rem}}$). Open symbols (above the line at $\chi /\chi_{0}\;=\;0$) represent the $\chi^{\prime \prime }$ component, while closed symbols (below $\chi /\chi_{0}\;=\;0$) are $\chi^{\prime }$ values. The inset presents the critical temperatures versus AC field amplitude (*h*) obtained for the GF specimen using different criteria. (**b**) Detail of magnetic moment versus DC applied field (*H*) curves taken at different temperatures (*T*) for the GF sample. The upper left inset presents the complete hysteresis loops for the GF sample, whereas the upper right inset shows the forth quadrant of the M(H) curves for the PF sample at different temperatures. The axis units are consistent throughout the panels.

Temperature-dependent AC susceptibility measurements were performed for two different specimens: the structured film with the central groove, named Grooved Film (GF); and a sister sample without the channel, identified as Plain Film (PF). This allows us to compare directly both responses and identify the influence of the WL. Figure 2a shows both the $\chi^{\prime }$ (closed symbols) and $\chi^{\prime \prime }$ (open symbols) components of $\chi_{AC}$. Data for the GF and PF samples are presented as colored and gray curves, respectively, for different excitation field amplitudes (*h*), f=100Hz, measured under the remnant DC magnetic field ($H_{\mathrm{rem}}$) of the superconducting magnet (∼1 Oe). Calibration of both components of the susceptibility was performed through division of the acquired data by the value of the real component of the susceptibility at its most negative plateau, $\chi_{0}^{\prime }$, at which the studied films are in the Meissner state.

For h=0.5Oe (square symbols), both samples show a sharp transition at 9.1K below which the $\chi^{\prime }$ and $\chi^{\prime \prime }$ components separate from each other. For the GF film, the transition shows a characteristic signature of granular systems: a double drop in the in-phase component $\chi^{\prime }$ together with a couple of peaks in $\chi^{\prime \prime }$ [52]. Differently, the PF specimen presents a single step accompanied by a single peak in $\chi^{\prime }$ and $\chi^{\prime \prime }$, respectively. For the structured sample the effective volume fraction of the pristine grains ($f_{g}$) is 0.92 [57,58], with an estimated superconducting transition width $\delta T_{c}=1.0\;$K. This quantity measures the difference between the onset of the transition and the point at which the curve reaches the Meissner plateau. On the other hand, the same transition is much narrower for the PF case with $\delta T_{c}$ = 0.6 K, defined when the $\chi^{\prime }$ reaches −0.995. For higher AC field amplitudes, the transition due to the pristine part of the sample becomes gradually broader as a consequence of a decreasing of its capacity to screen out the AC field. For instance, Figure 2a includes measurements for both films at *h* = 3.8 Oe (diamond symbols): transitions are broader than those recorded at smaller values of *h*, being even wider for the GF as compared to that for the PF. A similar effect was shown by Navau et al. [58]. The authors have numerically calculated the effects on the AC susceptibility of a WL between two square grains in a film with different granular fractions. In that case, the first transition for the specimen with two identical grains, i.e., when $f_{g}<1$, is wider than for the plain sample ($f_{g}=1$), which is similar to our specimen PF. Besides that, the magnitude of the intrinsic peak in $\chi^{\prime \prime }$ is also larger for the plain sample. These two features are in agreement with our experimental results. In other words, those effects are a consequence of the existence of a poorer superconductor between the grains, allowing the penetration of magnetic flux into their inner edges.

The inset in Figure 2a shows the dependence of $T_{c}$, due to the grains, on *h* evaluated at the first drop in $\chi^{\prime }\left(T\right)$ onset point and as it reaches half of its minimum value (mid-point). The weak-link critical temperature, $T_{c}^{WL}\left(h\right)$, defined by the second drop in $\chi^{\prime }\left(T\right)$ is also represented. The onset $T_{c}$ obtained for the various *h* differs only within experimental error, in agreement with the PF data, as the drive field amplitude is not large enough to influence the stronger intragrain shielding capacity. On the other hand, by comparing the mid-point critical temperatures for both grain and WL, a stronger variation with *h* is observed for the latter. This fact can be attributed to structural inhomogeneities in the groove, i.e., the presence of WLs for which the intergranular magnetic response is strongly affected by *h*, being consistent with the expected damage caused by the gallium ion beam, which is confined to such region.

Turning our attention now to DC magnetic measurements, a maximum in the magnetization hysteresis loop, M(H), is expected to occur in the decreasing field branch for negative near-zero applied fields as a consequence of the critical current density $J_{c}$ dependence on *H* [59]. The actual peak position can be estimated using critical-state models for different J(H) relations considering specific geometries [59,60] and it is known to be shifted toward H=0 as the sample thickness is decreased, with thin films as an extreme example [61,62]. Figure 2b shows a detail of the magnetization curves, M(H), for the GF and PF samples at different temperatures, while the upper left inset therein presents the complete hysteresis loop for the GF sample revealing the expected global maximum near H=0, also visible in the upper right inset for the PF specimen. In the main panel, local maxima in M(H) can be observed for all curves with peak positions moving away from zero as the temperature is decreased for the GF sample. Those local maxima are absent on the corresponding curves for the PF sample, as shown in the upper right inset. This feature has been observed previously for granular samples [62,63] and can be understood considering the specimen morphology and magnetic history. As the field is increased from H=0, flux penetrates and is pinned inside the grains. Once *H* is decreased from its maximum magnitude, the total magnetic field at a grain boundary is, therefore, a balance between opposite contributions, one from the applied field and the other resulting from the trapped flux lines inside neighboring grains. In thin films, a global peak in M(H) for H>0 is thus unambiguously related to granularity, resulting from a complex WL network, and appears as the field contributions cancel each other, leading to a null net magnetic field at the grain boundaries [63]. The fact that there is a local maximum for H>0 only in the grooved sample – besides a global one around zero field – is due to the presence of a single WL. A detailed investigation about this secondary maximum in M(H) will be reported elsewhere. Thus, based on all these evidences presented by its AC and DC magnetic responses, the structured film with the central groove behaves as two grains interconnected by a single WL. More recently, it has also been demonstrated that it is possible to actively control the vortex pinning potential between patterned insulating groove-like regions in Nb nanodevices by applying currents into the superconducting film from a normal metal [9].

Whereas the performed magnetic measurements, as a global technique, probe the volume-averaged magnetic moment, resistivity measurements detect the superconducting state in a more localized way, when one or more superconducting paths are established between the electrodes [64,65]. For this purpose, electric contacts were placed onto the GF sample, as illustrated in Figure 1, allowing us to detect the grain response, similar to a pristine specimen, as well as the SS′S contribution, at the vicinity of the groove.

**Figure 3:**
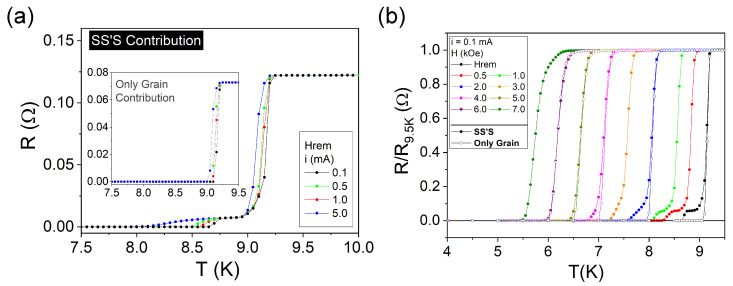
(Color online) (**a**) Temperature-dependent resistance curves for the SS′S contribution (main panel) and for the grain region (inset) for different applied currents ranging from 0.1 to 5.0 mA at $H_{\mathrm{rem}}$. (**b**) Temperature-dependent resistance normalized by the resistance at 9.5 K for the SS′S contribution (closed symbols) at *i* = 0.1 mA and different applied DC magnetic fields ranging from remnant field to 7 kOe, and for remnant field, 2 kOe, 4 kOe, 5 kOe, and 6 kOe for the grain region (open symbols).

Temperature-dependent resistance measurements, R(T), show a sharp superconducting transition at 9.2 K at remnant field for the plain region, as illustrated in the inset of Figure 3a. Although no noticeable difference on the onset $T_{c}$ was detected for applied currents ranging from i= 0.1 mA to i= 5.0 mA, the transition width $\delta T_{c}$ increases from 0.15 K for 0.1 mA to 0.20 K for all the other values of the current. The same transition as measured for the SS′S contribution, i.e., including the groove, is presented in the main panel of Figure 3a. As before, there is no important dependence of the grain $T_{c}$ on the applied current, but the data show an increase in $\delta T_{c}$ up to 0.30 K. Furthermore, a second transition, related to the groove, can be observed below 8.75 K. The mid-point WL critical temperature is shown to depend on the current as values obtained from the temperature-derivative of R(T) reveal that $T_{c}^{WL}$ = 8.70 K, 8.65 K, 8.55 K, and 8.25 K for i= 0.1 mA, 0.5 mA, 1.0 mA, 5.0 mA, respectively. The second transition width is as wide as 0.90 K for the highest value of applied current. At this point, one should be careful when comparing Figure 2a and Figure 3a. In the AC susceptibility curves, the AC driving magnetic field acts to probe the magnetic response of the specimen and, therefore, has a different physical role in the measurements than the applied DC magnetic field. Thus, the only possible comparison is between the curves taken at remnant DC magnetic field, for the lowest values of excitation, i.e., *h* = 0.5 Oe and *i* = 0.1 mA, respectively. In this case, the different values observed via magnetic and resistance measurements for the temperature of the WL transition are a consequence of the difference between the techniques. Particularly, a superconducting transition will be observed in an electrical transport measurement as soon as there is a percolative transport of supercurrent between the two electrodes. When decreasing the temperature from the normal state, this event tends to occur before the volume-average transition to superconductivity gauged in the magnetic measurements. It is also important to note that the resistance magnitude of the grain and SS′S contributions cannot be directly compared as the distances between the electrodes are different.

The evolution of the superconducting transition for the SS′S contribution with different applied fields at a fixed current *i* = 0.1 mA is shown in Figure 3b normalized by the resistance values at 9.5 K, $R_{9.5K}$, for each curve. The transition related to the grain contribution becomes wider and the onset $T_{c}$ decreases down to 6.50 K at *H* = 7 kOe. As the applied field increases and so does $\delta T_{c}$, it becomes difficult to differentiate between the first transition and the second one due to the groove. To visualize this trend, Figure 3b also presents the $R\left(T\right)/R_{9.5K}$ evolution for the grain only, i.e., between the electrodes 2 and 3, as shown in Figure 1b. At $H_{\mathrm{rem}}$, the second transition is well defined for the SS′S data, and it does not appear for the grain, as expected. This difference progressively becomes more subtle until 6 kOe, when it is not possible to resolve any vestige of the second transition. It occurs due to the overall deterioration of superconductivity of each contribution which leads to similar critical temperatures at this DC applied field.

From Equation (1) and the magneto-transport measurements, several normal-state and superconducting parameters for the grain and WL contributions were determined. The overall crystal quality was estimated by the Residual Resistivity Ratio (RRR), which is the ratio of the room-temperature resistivity to the resistivity at 10 K ($\rho_{300K}/\rho_{10K}$). RRR is a hallmark measurement of the degree of disorder in a given sample, as the residual resistivity is directly related to the concentration of defects. In this way, more defects lead to a higher $\rho_{10K}$, indicating a reduction of the electronic mean free path, and consequently a lower RRR. As expected, the value of RRR for the grain is higher than for the WL, as reported in Table 1. Moreover, although RRR for the grain may be comparable to other reported values for sputtered Nb films [66], it is considerably smaller than what is found for single-crystal Nb films [67]–this is an indication of the important role played by the polycrystalline morphology of the grain region in the resistivity response. It is also worth mentioning that for the SS′S contribution, i.e., the groove added to the neighbor grains, RRR = 6.47, similar to values reported by Dobrovolskiy et al. [18]. These values indicate a poorer quality for the WL due to the milling and implantation processes of gallium which lead to the increase of the concentration of defects on the grooved region. Furthermore, their onset critical temperatures, mean free paths, *l*, coherence lengths at 0 K, ξ(0), and penetration depth, λ(0), are shown in Table 1. The effects of milling the Nb film in the normal state properties are analysed ahead.

Based on features of the Fermi level for Nb, Mayadas et al. [68] reported a material constant relationship given by $\rho_{0}l$ = 3.72 $\times 10^{-6}$ μΩcm${}^{2}$, where $\rho_{0}$ is the sample residual resistivity. Using this equation and $\rho_{0}$ values obtained from the best fits of our ρ(T) data, which will be discussed later and are reported in Table 2, we were able to estimate the mean free path for the grain and WL regions. In the case of the value of $\rho_{0}$ obtained for the WL, it is important to keep in mind that the shape of the groove has been approximated in carrying out the resistivity calculations. This may lead to a slight misrepresentation of the actual value of $\rho_{0}$, which would impact the quantities to be obtained as follows. Nevertheless, the qualitative direct comparison between the grain and weak-link presently in study will hold true. As expected, *l* is much lower in the WL due to impurities introduced during the milling process, however, in both cases, it is reasonable [18] to consider that the sample is in the dirty limit, in which the mean electronic free path is smaller than the coherence length—a scale that defines the typical length within which the superconducting order parameter can vary appreciably. It is then possible to calculate the temperature dependent coherence length, ξ(T), in the dirty limit as [69]:

(2) $$\xi \left(T\right)=0.855\sqrt{\frac{\xi_{0}l}{1-T/T_{c}}},$$

where $\xi_{0}$ is the so-called Pippard’s coherence length and is of the order of 39 nm for Nb [64]. The results presented in Table 1 evidence a significant suppression in ξ(0) in the WL, highlighting the detrimental effect of its presence in the sample superconducting properties.

Another important length scale present in superconductors is the penetration depth λ(T), which represents the typical distance magnetic fields are able to penetrate the material. Considering the dirty limit, it can be calculated in terms of the mean free path as [69]:

(3) $$\lambda \left(T\right)=0.613\lambda_{L}\left(0\right)\sqrt{\frac{\xi_{0}}{l\left(1-T/T_{c}\right)}},$$

where $\lambda_{L}\left(0\right)$ is the London penetration depth at *T* = 0, also of the order of 39 nm [70]. The value obtained for λ(0) for the grain after this procedure is listed in Table 1. It is somewhat smaller than other typical values found in the literature for Nb thin films of similar thickness [71]. Nevertheless, an increase in λ(0) is observed for the WL compared to the grain. This direct comparison indicates a lower ability of the WL to shield the magnetic field due to the suppression of superconductivity in this region.

### 3.3. Normal State Properties

The resistivity curves were analyzed in the framework of the Boltzmann transport, Bloch-Grüneisen, and Wilson-Grüneisen theories [28,29,31,72,73]. The fundamental equation to describe the electron-phonon (e-ph) interaction in the temperature-dependent resistivity is presented as the second term on the right side of Equation (4),

(4) $$\rho \left(T\right)=\rho_{0}+\rho_{e-\mathrm{ph}}=\rho_{0}+K_{0}{\left(\frac{T}{\Theta_{D}}\right)}^{n}\int_{0}^{\Theta_{D}}\frac{x^{n}}{\left(e^{x}-1\right)\left(1-e^{-x}\right)}dx$$

where $\rho_{0}$ is the residual resistivity due to defect scattering and $\Theta_{D}$ = 275 K for Nb [74].

The exponent *n* depends on the nature of the interaction and usually assumes the values 2, 3, or 5. When n=3, the main mechanism is called sd interband scattering and the resistivity contribution is labelled $\rho_{sd}$. This mechanism is a known feature of transition metals where conduction band electrons with a high Fermi velocity (*s* band) can be scattered by lattice vibrations into an unfilled *d* band with a low Fermi velocity and vice-versa. For a single band metal, however, the most important mechanism arises from phonon-scattered electrons within the *s* shell, named ss intraband scattering. In this case, n=5 and this contribution is labelled $\rho_{ss}$.

**Figure 4:**
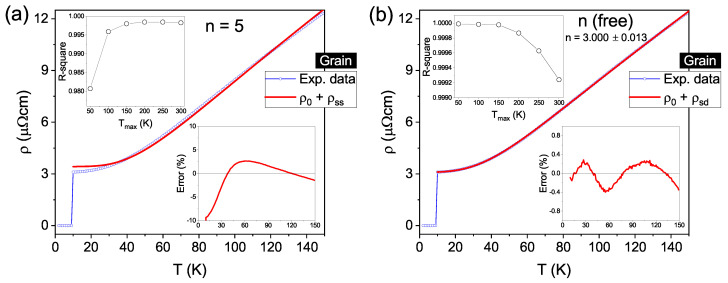
(Color online) Temperature-dependent resistivity for the grain contribution. In panel (**a**), the red line is the fitting for n=5 in Equation (4) up to 150 K, whereas (**b**) shows the fitting for *n* as a free parameter for same equation. The upper and lower insets for both panels show the R-square coefficient versus the upper limit of temperature $T_{\max}$ and the relative fit error versus temperature, respectively.

Based on Ref. [18], an exponent n= 5 was chosen for the plain grain contribution and an attempt to determine the upper limit of temperature ($T_{\max}$) for which $T\ll \Theta_{D}$ was made. It is worth mentioning that for temperatures near $\Theta_{D}$, the contribution $\rho_{e-\mathrm{ph}}$ is proportional to *T* regardless of the value of *n* [75]. As the resistivity measurements were carried out up to room temperature, different curves were fitted by changing the maximum temperature in steps of 50 K, from 50 K up to 300 K, always considering the initial point at 10 K, i.e., above $T_{c}$. The upper inset of Figure 4a illustrates the R-square coefficient for the fitting curves against $T_{\max}$. This analysis reveals a clear enhancement of R-square up to $T_{\max}$ = 150 K, above which point it stays nearly constant, i.e., three decimal places are equal. Such behavior deviates from the expected $T\ll \Theta_{D}$ criterion, which implicates that higher maximum temperatures would lead to a worst fit of Equation (4). Nevertheless, the main panel of Figure 4a exhibits the experimental data in blue and the fitting curve in red, obtained by setting $T_{\max}$ as 150 K to present an example. It fits reasonably well for intermediate temperatures, but clearly deviates for lower ones, as revealed by the bottom inset. One can observe the relative fit error, defined as [($\rho_{\mathrm{measured}}-\rho_{\mathrm{fit}}$)/$\rho_{\mathrm{measured}}\;\times$ 100%], which shows a minimum close to −10% at lower temperatures, increasing up to a maximum value of ∼3% and decreasing gradually for higher temperatures. A similar behavior appears if we choose values for $T_{\max}$ larger than 150 K.

Although a substantial difference appears between the data below 30 K, a relatively high value of R-square is reached when taking *n* = 5, as given in Table 2. To find a solution for such a discrepancy, in the next step, the exponent *n* was left as a free parameter in the fitting procedure. Again, the R-square coefficient evolution with different $T_{\max}$ values was investigated. The results in the upper inset of Figure 4b reveal an almost constant, close to 1, behavior up to 150 K, decreasing above it – as expected when $T\to \Theta_{D}$. As such, $T_{\max}$ = 150 K is found to be the highest value of maximum temperature satisfying the $T\;\ll \;\Theta_{D}$ criterion. The fit of Equation (4) for such $T_{\max}$ yields an exponent n=(3.000±0.013) and an R-square value considerably higher than those obtained for *n* = 5, as shown in Table 2. Furthermore, the main panel of Figure 4b shows both the measured and the new fitting curves, evidencing a greater agreement between them at low temperatures. The relative fit error, depicted in the lower inset, is less than ±0.4% over the whole temperature range. Therefore, although there is an undeniably significant contribution to the electronic scattering due to the grain morphology, the previous analysis demonstrates that the temperature-dependent resistivity of the plain Nb film (grain) is governed by the sd interband scattering mechanism, instead of the ss intraband one. This behavior has also been previously observed for high-purity Nb bulk samples [32] and sputtered Nb films [66].

**Figure 5:**
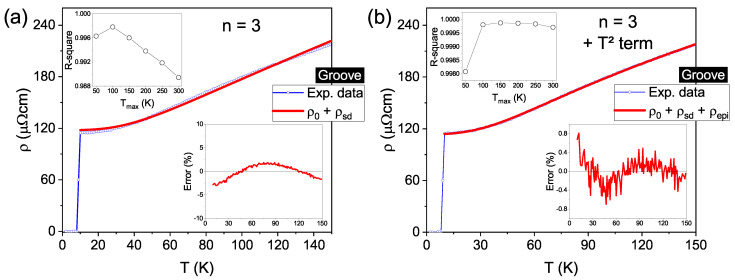
(Color online) Resistivity versus temperature (up to 150 K) curves for the groove. The fitting depicted by the red curve in (**a**) represents the sd band scattering (n=3), as described by Equation (4). In (**b**), the red curve represents the fitting considering an additional contribution given by Equation (5) in comparison to the fitting in (**a**). The lower inset in both panels represents the relative fit error and the upper insets shows the quality of the fit given by the R-square coefficient, which changes when the temperature upper limit is varied.

Concerning the resistivity data for the grooved region, Equation (4) was used in the first fittings. We assumed the exponent *n* = 3 based on the results discussed in the previous paragraph for the plain film region. Nonetheless, the fitting curve shows a significant deviation from the experimental data, which can be as high as 3%, over the whole temperature range, as presented in the main panel of Figure 5a and reinforced by the relative fit error in the bottom inset. The R-square coefficient versus $T_{\max}$ curve in the top inset shows a maximum value at 100 K, which is still far from ideal fitting. It should be pointed out that the previously mentioned possible misrepresentation of $\rho_{0}$ due to the approximation of the groove shape would only contribute to the ρ(T) curve as a constant. Therefore, this is not the cause for the large error obtained as it does not influence the variation in ρ(T) as the temperature increases. In other words, the behavior of the temperature-dependent resistivity is correctly represented in Figure 5. In fact, an additional scattering mechanism must be considered, due to the gallium ions implanted during the notch excavation. For highly disordered metals, it is necessary to account for the quantum interference of all different possible sources of electron scattering, for which the overall effect may result in the presence of a $T^{2}$ term in ρ(T) [35,76]. Doing so has led to the development of the so-called electron-phonon-impurity (epi) interference effect, which also considers the inelastic scattering of electrons by impurities. It has been shown experimentally that such a theory successfully describes the resistivity behavior of several metallic thin films, including Nb, over a large range of temperatures [77,78,79]. To account for the presence of the epi mechanism in the groove, an additional term, given by Equation (5), was added to Equation (4), in accordance with Matthiessen’s rule. It is noteworthy that at low temperatures ($T<\Theta_{D}/5$), the integral in Equation (5) approaches $\pi^{2}/6$, and its temperature dependence is simplified to $\rho_{0}BT^{2}$ [76,77], however, this approximation is not used here.

(5) $$\rho_{\mathrm{epi}}\left(T\right)=\rho_{0}BT^{2}\left(\frac{6}{\pi^{2}}\right)\int_{0}^{\left(\frac{\Theta_{D}}{T}\right)}\left[\frac{x^{2}e^{x}}{{\left(e^{x}-1\right)}^{2}}-\frac{x}{\left(e^{x}-1\right)}\right]dx$$

where *B* is the epi coefficient.

Figure 5b illustrates the resistivity fit considering the terms $T^{3}$ and $T^{2}$. The main panel shows a good agreement between the measured and fitting curves over the full range of temperature, which is confirmed by the bottom inset presenting a maximum fitting error value of 0.8%. Besides that, as shown in the top inset and listed in Table 2, the R-square coefficient goes through a maximum at 150 K, a value perfectly compatible with that arising from the previous analysis for plain Nb. Therefore, the effect of Ga impurities—implanted into the groove during the FIB excavation—on the temperature-dependent resistivity, manifests itself as an additional scattering term (epi), properly represented by the expression in Equation (5).

### 3.4. The Evolution of the Critical Temperature with Disorder

In the last sections, the effects of FIB milling on the superconducting and normal state properties of the tailored Nb film prepared using a dose of 1.2 nC/μm${}^{2}$ were demonstrated in detail. The changes in the critical temperature of the WL region, $T_{c}^{WL},$ and the emergence of the defect-driven quadratic-in-*T* term in ρ(T) are both attributed to the presence of implanted Ga impurities in the groove region. Recently, the mechanism behind the simultaneous emergence of both effects in superconducting thin films has been elucidated as resulting from the distortion and softening of the lattice due to stabilized defects [37], as the ones due to the FIB milling process. The theory predicts that strong-coupled superconductors, such as Nb, present a decrease in $T_{c}$ as the residual resistivity $\rho_{0}$ is increased—which arises by increasing the density of defects in the specimen. An analytical expression that allows one to gauge the variation of $T_{c}^{WL}$ for the grooved Nb samples has been derived as [37]:

(6) $$\frac{\delta T_{c}^{WL}}{T_{c0}^{WL}}\approx -t_{1}\left(\frac{\delta \rho_{0}}{\rho_{00}}\right)-t_{2}{\left(\frac{\delta \rho_{0}}{\rho_{00}}\right)}^{2},$$

where $T_{c0}^{WL}$ and $\rho_{00}$ are the values of $T_{c}^{WL}$ and $\rho_{0}$ for the lowest available concentration of defects, $\delta \rho_{0}=\rho_{0}-\rho_{00}$, and $\delta T_{c}^{WL}=T_{c}^{WL}-T_{c0}^{WL}$. The positive coefficients $t_{1}$ and $t_{2}$ are related to different electronic and phononic material characteristics as discussed in Ref. [37].

To investigate this trend for this kind of nanofabricated single WL samples, we varied the dose used during the FIB milling from 0.1 nC/μm${}^{2}$ to 1.8 nC/μm${}^{2}$. $T_{c}^{WL}$ values were then evaluated by the mid-point of the weak-link transition in *T*-dependent AC susceptibility curves, such as those presented in Figure 2a. Although our measurements do not allow access to $\rho_{0}$ values, it is true that the residual resistivity is directly related to defect concentration, being thus reasonable [25,80,81,82,83,84] to use the dose as the relative control parameter gauging the amount of impurities introduced in the film by the incoming Ga atoms. In other words, we may assume the relationship $\delta \rho_{0}/\rho_{00}∼\delta \left(\mathrm{dose}\right)/{\mathrm{dose}}_{0}=\delta \left({\mathrm{dose}-\mathrm{dose}}_{0}\right)/{\mathrm{dose}}_{0}$, with dose${}_{0}$ = 0.1 nC/μm${}^{2}$. Accordingly, $T_{c0}^{WL}$ represents the WL transition temperature for the sample with dose${}_{0}$.

**Figure 6:**
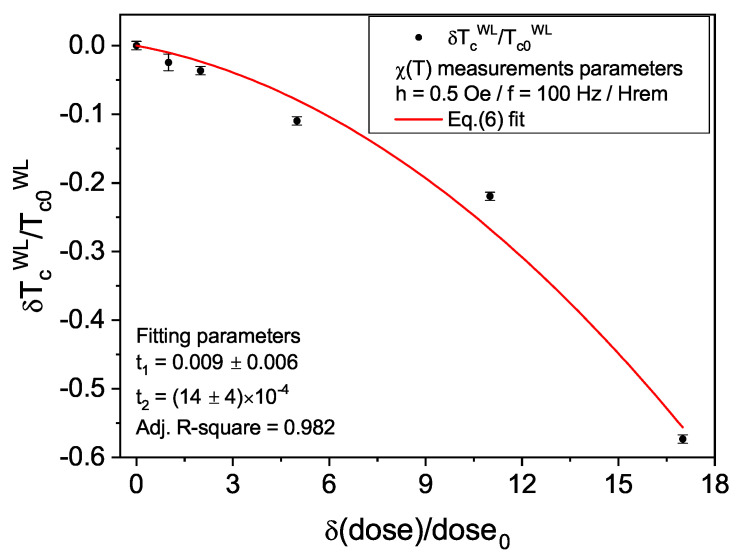
Relative change of the critical temperature as a function of the relative beam dose used during groove milling. Red solid curve is the fit of Equation (6) using the FIB dose as control parameter. This analysis demonstrates that the data follows the expected trend for strong-coupled superconductors.

The evolution of the relative $T_{c}^{WL}$ variation with the concentration of defects measured by the relative dose variation is presented in Figure 6. The first clear characteristic is the presence of the expected monotonous decrease of $T_{c}^{WL}$ as the defect concentration is increased, reaching values as low as (3.5 ± 0.1) K. Furthermore, Equation (6) was fitted to the data only by replacing $\rho_{0}$ by the values of the respective doses used in the fabrication process. The model agrees reasonably well with the experimental data, despite the values of $T_{c}^{WL}$ being collected via volume-average magnetic measurements, and not from resistivity. The overall agreement seems to validate the relationship assumed to exist between $\delta \rho_{0}$ and δ(dose). This not only corroborates the findings of [37], but also indicates that the model is a rather robust description of the underlying mechanism leading to the simultaneous occurrence of a variation in $T_{c}$ and the emergence of the quadratic-in-*T* resistive contribution in defect-bearing specimens. The relationship between superconductivity and normal state properties in our single WL Nb samples is then demonstrated to arise from the inclusion of defects, evidencing once again the milling procedure as the cause of the deterioration of superconducting properties.

## 4. Conclusions

We have successfully fabricated a thin-film granular system, composed by two high-quality Nb grains separated by a single artificial weak-link excavated using the FIB technique. This sample allowed us to gauge the effects of granularity in the most simple system possible, considering materials and etching technique widely used to obtain nanopatterned superconducting devices. The analysis of the AC and DC magnetic responses evidenced a couple of features related to its granular morphology, namely a double-step transition as the sample is cooled down from the normal to the Meissner state and the existence of a local maximum in the hysteresis loop. Furthermore, transport measurements also revealed the existence of a double transition to the superconducting state, caused by the presence of the groove in the center of the film. By isolating the grain and groove contributions to the resistivity, we were able to determine several normal state and superconducting parameters related to each of them. These results demonstrate a slight worsening of the sample performance caused by the notch. Furthermore, we conducted a careful investigation of the normal state scattering mechanisms in the different parts of the sample by fitting the temperature-dependent resistivity curves based on the Wilson-Grüneisen model. Thus, it was shown that for the plain Nb thin film sd interband scattering is the main mechanism contributing to the resistivity. When studying the behavior for the groove region, however, an additional defect-driven quadratic-in-*T* term arising from the presence of Ga impurities implanted during the FIB excavation was shown necessary to describe properly the scattering process in the sample. This effect is shown to be correlated to a decrease in the superconducting critical temperature by analyzing different samples produced with varying degrees of disorder, being in a good agreement with a recent theoretical description.

## Figures and Tables

**Table 1:** Resistivity at 300 K and 10 K; RRR; Superconducting critical temperature, $T_{c}$; mean free path, *l*; coherence length, ξ(0); penetration depth, λ(0). All listed parameters were obtained for the grain and weak-link contributions.

Parameters	Grain	Weak-Link
$\rho_{300}$ [μΩcm]	22.48	302.49
$\rho_{10}$ [μΩcm]	3.11	114.62
RRR	7.23	2.64
$T_{c}^{onset}$[K]	9.25±0.05	8.75±0.05
*l* [nm]	12.02±0.05	0.33±0.03
ξ(0) [nm]	18.51±0.04	3.06±0.14
λ(0) [nm]	43.06±0.09	259±12

**Table 2:** ρ(T) fittings for the grain and groove components.

Contribution	ρ(T)=	n	$\rho_{0}$ (μΩcm)	*B* ($10^{-5}$ K${}^{-2}$)	$K_{0}$ (μΩcm)	Adj. R-Square
Grain	$\rho_{0}+\rho_{ss}$	5	3.426 ± 0.018	-	80.5 ± 0.3	0.99799
Grain	$\rho_{0}+\rho_{sd}$	3.000 ± 0.013	3.095 ± 0.004	-	39.21 ± 0.25	0.99998
Groove	$\rho_{0}+\rho_{sd}$	3	117.8 ± 0.3	-	437 ± 2	0.99601
Groove	$\rho_{0}+\rho_{sd}+\rho_{epi}$	3	113.30 ± 0.09	3.93 ± 0.06	307 ± 2	0.99988

## Data Availability

Data is contained within the article.

## Sample Availability

Nb thin films are available from the authors.

## Abbreviations

The following abbreviations are used in this manuscript:

FIB	Focused ion beam
MO	Magneto-optical
AFM	Atomic force microscopy
EDS	Energy Dispersive X-ray Spectrometry
SEM	Scanning Electron Microscope
GF	Grooved Film
PF	Plain Film
S	Plain region of Grooved Film
S′	Groove region of Grooved Film
$T_{c}$	Superconducting critical temperature
WL	Weak-link
$T_{c}^{WL}$	Weak-link critical temperature
$H_{\mathrm{rem}}$	Remnant DC magnetic field
*h*	Applied excitation field amplitude
*T*	Temperature
ρ(T)	Temperature-dependent resistivity
*n*	Power-law exponent in ρ(T)
$A_{S}$ ($A_{S^{\prime }}$)	Cross-section area of the grain (groove)
$I_{S}$ ($I_{S^{\prime }}$)	Length of the grain (groove)
$\rho_{0}$	Residual resistivity
$\rho_{S^{\prime }}$	Groove resistivity
$\rho_{G}$	Grain resistivity
$\rho_{epi}$	Electron-phonon-impurity resistivity contribution
$\rho_{sd}$	sd interband scattering resistivity contribution
$\rho_{ss}$	ss intraband scattering resistivity contribution
$\rho_{10}$	Resistivity at 10 K
$\rho_{300}$	Resistivity at 300 K
$V_{G}$	Voltage measured between electrodes 2 and 3 (plain region)
$V_{\mathrm{SS}\prime S}$	Voltage measured between electrodes 3 and 4 (SS′S contribution)
*B*	Electron-phonon-impurity coefficient
$\Theta_{D}$	Debye temperature
$T_{\max}$	Upper limit of temperature to fit ρ(T)
$\chi_{AC}\left(T\right)$	Temperature-dependent AC susceptibility
$\chi^{\prime }$	Real part of $\chi_{AC}$
$\chi^{\prime \prime }$	Imaginary part of $\chi_{AC}$
*f*	Frequency
*R*	Resistance
RRR	Residual resistivity ratio
*l*	Mean free path
ξ	Coherence length
λ	Penetration depth
$H_{c2}$	Upper critical field
